# Self-Persuasion Increases Healthy Eating Intention Depending on Cultural Background

**DOI:** 10.3390/ijerph17103405

**Published:** 2020-05-13

**Authors:** Shuang Li, Cor van Halen, Rick B. van Baaren, Barbara C. N. Müller

**Affiliations:** 1 Institute of Governance, Shandong University, Qingdao 266237, China; 2 School of Politics and Public Administration, Shandong University, Qingdao 266237, China; 3 Behavioural Science Institute, Radboud University, 6500 HE Nijmegen, The Netherlands; c.vanhalen@psych.ru.nl (C.v.H.); r.vanbaaren@psych.ru.nl (R.B.v.B.); b.muller@bsi.ru.nl (B.C.N.M.)

**Keywords:** self-persuasion, direct persuasion, cultural background, healthy eating intention, persuasion communication

## Abstract

Unhealthy eating behavior has become a global health risk and thus needs to be influenced. Previous research has found that self-persuasion is more effective than direct persuasion in changing attitudes and behavioral intentions, but the influence of the cultural backgrounds of those being persuaded remains unclear. We conducted two studies to investigate the effectiveness of self-persuasion and direct persuasion techniques in promoting healthy eating intention among different ethnicities in the Netherlands. Native Dutch, Moroccan–Dutch, and Turkish–Dutch participated both online and offline. Participants saw a poster with either a self-persuasion message (“Why would you choose healthier food?”) or a direct persuasion message (“Choose healthier food!”), and were then asked to report their intention to eat healthily in the upcoming month. Significant cultural differences were found between native Dutch and Moroccan–Dutch in Study 1, and between the native Dutch and Turkish-Dutch who participated offline in Study 2. Accordingly, cultural background was found to moderate the relationship between persuasion and healthy eating intention among these groups. These results provided preliminary evidence for the moderation effect of persuasion on healthy eating intention: Self-persuasion appears to be more effective for people with an individualistic background, and direct persuasion appears to be more effective for people with a collectivistic background.

## 1. Introduction

Unhealthy eating behavior has become one of the most recognized global health risks, and has been identified as a major cause of a number of severe physical and mental health problems, such as cancer, insomnia, and depression [1,2]. According to a report from the Dutch National Institute for Public Health and the Environment [3], the Dutch population ranks quite high in consumption of high-calorie food, such as snacks and fats, and ranks relatively low in consumption of vegetables, fish, and fruit in comparison to 12 other European countries.

In the Netherlands, much effort has been made to discourage unhealthy eating behaviors, for instance the 5-year nationwide mass media campaign on the prevention of being overweight, launched in 2002 [4], and the well-known public campaign “Gezonder Mag” (translation: “Healthier is allowed”) [5]. However, these public campaigns rarely took the audience’s cultural background into account. Like many Western countries, the Netherlands have a culturally diverse population as a result of a massive influx of immigrants worldwide after World War II. In January 2018, up to 23% of the national population consisted of residents with a migration background, the majority of them (around 60%) being immigrants from non-Western descent, mainly Turkish and Moroccan [6]. Moreover, a high prevalence of obesity has been observed in these two groups of immigrants [7,8], and research has showed that they have been faced with a more significant challenge to build a healthy lifestyle compared to native Dutch [9]. The different cultural backgrounds between native Dutch and these immigrant groups may lead to different susceptibility to persuasion techniques that are typically being used in health campaigns. Research has demonstrated the importance of taking individual susceptibility into account when applying specific persuasive techniques of influence. For instance, Kaptein and colleagues [10] found that individual susceptibility to Cialdini’s 6 persuasive principles [11] is related to one’s compliance to a persuasive request. Moreover, the correlation between susceptibility and compliance differed depending on which persuasive strategies were used, such that the relation was stronger when the consensus principle was applied. In another study, researchers found that persuasive messages that are tailored based on individual susceptibility led to a higher decrease in snacking consumption compared to messages that are not tailored [12]. This evidence supports the importance of using specific persuasive strategies according to one’s susceptibility, and the importance of personalizing persuasive messages in increasing its effectiveness. Therefore, tailoring persuasive messages according to individuals’ cultural backgrounds may greatly enhance the effectiveness of persuasion. Interestingly, until now, cultural background has rarely been taken into account in health campaigns which target Dutch citizens including local Dutch, Moroccan–Dutch, and Turkish Dutch. The current research investigated which role cultural background plays in the relationship between persuasion and healthy eating, and explored effective persuasive techniques to design health messages for these three sub-populations in the Netherlands.

Persuasion has long been used to elicit desirable changes in attitudes and behaviors across various domains, such as in decreasing cigarette consumption [13], promoting physical activity [14], and encouraging prosocial or sustainable behavior [15,16]. Traditionally used, direct persuasion targets people’s attitudes or behaviors through direct communication [17]: People are directly told what they should or should not do. Such an approach has been found ineffective and only capable of causing a short-term attitude change, if any [18]. Furthermore, direct persuasion could lead to a boomerang effect, for example resulting in an increased degree of smoking [19], presumably because this direct approach tends to trigger the message recipients’ psychological reactance and induce defensive responses [20,21,22]. 

A subtler persuasive technique is self-persuasion, which enables people to generate their own arguments towards a specific topic [18]. Self-persuasion can be induced in various ways, for instance by asking people to give a public speech about the benefits of the target behavior [23] or by asking questions which can trigger thoughts in favor of the target behavior [13]. Among others, the least costly and most straightforward way is presenting people with open-ended questions [24]. The assumption behind this practice is that arguments would be automatically generated when people are exposed to open-ended questions. A recent study affirmed this assumption by exposing participants to anti-alcohol posters formulated either as an open-ended question or as a statement, and then asking them to list their thoughts during the exposure to the posters [25]. It was found that participants who were exposed to the question-formulated poster indeed reported pro-arguments in response to the question.

Researchers state that the self-generated arguments produced in the self-persuasion process are more persuasive, compelling, and tailored to oneself, compared to the messages provided through direct persuasion [26,27]. In addition, self-persuasion can successfully avoid psychological reactance by making people both the source and target of persuasion [28,29]. 

Although self-persuasion has shown promising results in terms of its effectiveness of influence, we proposed that it is not a one-size-fits-all approach for culturally diverse target groups. The crux of self-persuasion is that it appeals to one’s sense of personal autonomy, whereas direct persuasion tries to induce compliance to requests of external parties from a feeling of social obligation [30]. This relates to critical cultural differences between the Dutch culture and the heritage culture of Moroccan and Turkish immigrants. Firstly, these groups are supposed to differ in individualism and collectivism, which reflect individuals’ values on personal autonomy and group interest [31]. As a prototypical Western culture, Dutch culture is more individualistic and independence-oriented [32]. As a result, native Dutch are supposed to prioritize their own needs and to value autonomy in making choices. In comparison, Moroccan and Turkish cultures are more collectivistic and interdependence-oriented. Its members typically focus more on others’ opinions and maintain life satisfaction by fulfilling obligations and complying with social norms [33,34,35]. In this vein, Moroccan–Dutch and Turkish–Dutch should be more likely to comply with direct persuasive messages in order to conform their behavior to social and normative expectations. On the contrary, native Dutch should be more susceptible to change their behavior through self-persuasion since they value personal autonomy. In addition, individuals’ susceptibility to persuasion is likely to be influenced by power distance, which reflects the extent to which the cultural members accept social hierarchy [35]. Mediterranean cultures, such as the Moroccan and Turkish, are traditionally more hierarchy-oriented, where people accept and respect orders from authorities [36]. By being used to situations in which one has to take a subordinated role, members of these cultures should act more readily to directive orders, such as direct persuasion commands, and experience less reactance.

Furthermore, the effectiveness of self-persuasion and direct persuasion was found to be regulated by individuals’ experienced agency, meaning that self-persuasion (vs. direct persuasion) works conditionally for individuals who experience high (vs. low) agency [37]. Coupled with this finding, research showed that adolescents with a non-Western cultural background experience less personal choice and behavioral autonomy compared to their Dutch counterparts [38]. This again supports our proposal that self-persuasion would be more successful to stimulate healthy eating intention in native Dutch, whereas direct persuasion would be more successful in Moroccan–Dutch and Turkish–Dutch. Two studies were performed to test this hypothesis, with Study 1 conducted among native Dutch and Moroccan–Dutch, and Study 2 among native Dutch and Turkish–Dutch. 

## 2. Study 1: Materials and Methods 

Design. A 2 (persuasion: Self-persuasion vs. direct persuasion) × 2 (cultural background: Dutch vs. Moroccan) between-subjects design was used, with persuasion and culture as factors, and healthy eating intention as the dependent variable. 

Sample. The experiment was initially programmed into an online survey questionnaire in Qualtrics and took approximately 10 min to finish. To recruit native Dutch and Moroccan–Dutch participants, we disseminated the survey link on Facebook pages, in several Moroccan Facebook groups, and asked friends and colleagues to help disseminate the link online within their social networks. People who were willing to participate could directly enter the survey by clicking the link. These participants did the survey questionnaire online in their own environment. However, the number of Moroccan–Dutch participants was not sufficient due to difficulties in recruiting this specific group. To obtain a comparable number of participants for both groups, the experimental materials were later printed out and brought to the center of Utrecht City: The researchers first roughly recognized people with Moroccan appearance, which was characterized by a dark skin tone, brown or black hair, and dark beards (for males). Then the researchers approached these people, asked whether they have a Moroccan background, and invited those who answered “yes” to complete the paper questionnaire on the spot. In the end, 115 participants took part in the experiment, of which 24 participants were excluded, because they did not complete the experiment (*n* = 8), indicated their origin as neither the Netherlands nor Morocco (*n* = 7), did not identify themselves with either culture (*n* = 4; see more details in “Procedure and materials”), or scored 2.5SDs above or below the mean on Individualism and Collectivism Scale [39], which was used to test participants’ cultural background in the current study (*n* = 5). As a result, 49 native Dutch and 42 Moroccan–Dutch were included in the final analyses (*M*_age_ = 20.51, *SD*_age_ = 2.61; *n*_female_ = 56, *n*_male_ = 35). All 49 native Dutch participated online, whereas 15 Moroccan–Dutch participated online and 27 Moroccan–Dutch offline^1^ (see Table 1 for participants’ demographics). All participants participated voluntarily and did not receive any compensation.

Procedure and materials. All the experimental materials were incorporated into a questionnaire written in Dutch. In the instructions, participants were told a cover story that the questionnaire consisted of two unrelated parts: The first part aimed to examine whether people with different cultural backgrounds perceive advertising differently, and the second part was a general health survey which aimed to know citizens’ health status. Before participants could start to complete the questionnaire, they were informed that they could withdraw their participation at any time without giving any reason, and had to give active consent. The study was conducted according to the principles expressed in the Declarations of Helsinki, and according to the guidelines of the institutional review board. Ethical approval was not required at the time of data collection (2016) by the institution’s guidelines and national regulations, as the research was not of a medical nature and there were no potential risks to the participants.

The first part of the survey asked for the participants’ age, gender, educational level, and cultural background. To access participants’ cultural background, they were asked to indicate their ethnic origin, native language, religion, origin of the participant’s father, and origin of the participant’s mother. For each item, a 0 was given if the answer indicated a Moroccan background (e.g., reporting origin as Morocco, native language as Arabic or Berber, and religion as Islam). A 1 was given if the answer indicated a Dutch background (e.g., reporting origin as the Netherlands, native language as Dutch, and religion as Christian or ‘no religion’). If participants gave irrelevant answers to one of the five items (e.g., declaring oneself or one of their parents to be German), they were excluded from the data analysis. For the remaining participants, a total score was calculated by adding up the scores on the five items, ranging from 0 to 5. Participants with a total score of 0 or 1 were categorized as having a Moroccan background, whereas those with a total score of 4 or 5 were categorized as having a Dutch background. Participants with a total score of 2 or 3 were further excluded since they did not clearly identify with either culture. 

Subsequently, participants’ individualism and collectivism levels were measured by using the 16-item Individualism and Collectivism Scale [40]. This instrument has the advantage of measuring the individualism–collectivism orientation in combination with power distance. It consists of four subscales: Horizontal individualism (Cronbach’s α = 0.65), which emphasizes self-reliance and distinction from groups without pursuing high status; vertical individualism (Cronbach’s α = 0.47), which emphasizes uniqueness from individual competitions and the pursuit of high status; horizontal collectivism (Cronbach’s α = 0.62), which emphasizes common goals with others, communal sharing, interdependence, and sociability without submitting to authority; vertical collectivism (Cronbach’s α = 0.74), which emphasizes the integrity of the in-group and group benefit by submitting to the will of group authorities. Participants had to answer these questions on a 9-point Likert scale (1 = “not at all agree”, 9 = “completely agree”). 

After the individualism and collectivism measure, participants were randomly assigned to either the self-persuasion condition or the direct persuasion condition. In both conditions, they were presented with a poster that was introduced as an advertisement [see similar procedures in 25]. An image was used in both posters: A well-built person holding an apple standing next to an obese person holding a hamburger. However, the image was accompanied by different messages. In the self–persuasion condition, the message was formulated as an open question: “Why would you choose healthier food?”, whereas in the direct persuasion condition, the message was formulated as a directive statement: “Choose healthier food!” 

Participants then received a filler task in which we asked them to evaluate the “advertisement” with seven items, such as the use of color and the position of the text. Subsequently, participants completed the “general health survey” on a 7-point Likert scale (1 = “totally disagree”, 7 = “totally agree”). First, they had to answer three filler questions about willingness to drink less alcohol in the upcoming month. 

Following the filler questions about drinking, three items adapted from an existing measurement [39] were used to measure healthy eating intention (e.g., “I want to eat more healthily in the upcoming month”). A higher total score indicated a healthier eating intention. Cronbach’s α of this scale was 0.93 in this study. 

In the end, participants were thanked and debriefed face-to-face or via email. All the materials and data of the current research are stored on the university’s file server in accordance with the institutional data management policy and can be accessed on OSF via https://osf.io/vsu5d/?view_only=b06ea55be6434f14b6fef60ac30029fd.

## 3. Study 1: Results

The statistical analyses were conducted in SPSS 25. Firstly, a MANOVA between native Dutch and Moroccan–Dutch on the four cultural dimensions was performed. Using Pillai’s trace, the multivariate testing showed a significant effect of cultural background, *V* = 0.48, *F*(4,86) = 19.88, *p* < 0.001, η_p_^2^ = 0.48. Univariate testing showed that cultural background was significant on all the variables (maximum *p* = 0.016, minimum η_p_^2^ = 0.06) except for vertical individualism (*p* = 0.133, η_p_^2^ = 0.03). See Table 2 for descriptions.

**Figure 1:**
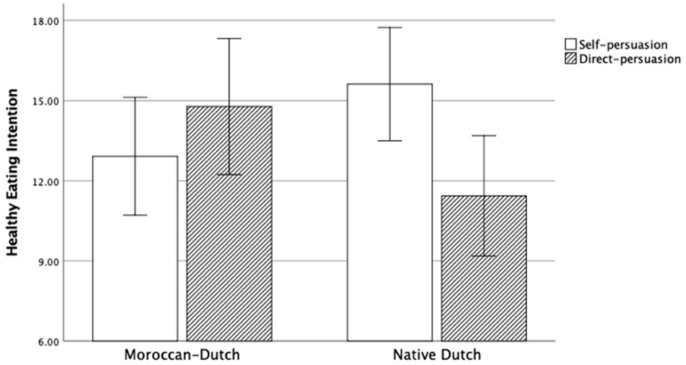
The interaction between persuasion and cultural background in Study 1.

An ANOVA was conducted on healthy eating intention, with persuasion and cultural background as two factors. Both the main effects of persuasion and of cultural background were not significant, *p*_persuasion_ = 0.316, *p*_culture_ = 0.780. A significant interaction was found between persuasion and cultural background, *F*(1,87) = 6.90, *p* = 0.010, η_p_^2^ = 0.07 (see Figure 1). Simple effect analyses were conducted: Dutch participants in the self-persuasion condition reported a significantly higher intention on healthy eating (*M* = 15.62, *SD* = 4.06) than in the direct persuasion condition (*M* = 11.43, *SD* = 5.70), *F*(1,87) = 7.22, *p* = 0.009, η_p_^2^ = 0.08. However, the healthy eating intention reported by Moroccan–Dutch participants did not significantly differ between the self-persuasion condition (*M* = 12.92, *SD* = 6.42) and the direct persuasion condition (*M* = 14.78, *SD* = 5.40), *F*(1,87) = 1.21, *p* = 0.275, η_p_^2^ = 0.01.

As expected, we found a significant interaction between cultural background and persuasion on healthy eating intention. Consistent with previous findings in other health domains [13,25], self-persuasion was found to be more effective than direct persuasion for native Dutch. However, the two types of persuasion did not show a significantly different effect for Moroccan–Dutch, though the average mean did show a difference in the expected direction. This non-significant effect could result from acculturation [41] or low statistical power. Although we only found support for our hypothesis in Dutch participants, it provided preliminary evidence that cultural background moderates the persuasion effect.

Results on the two collectivist dimensions supported the presumed cultural differences between native Dutch and Moroccan–Dutch. In line with cultural psychological literature [31], the Moroccan-Dutch participants showed a higher degree of horizontal and vertical collectivism than the native Dutch participants, indicating Moroccan–Dutch are relatively more interdependent and family-oriented. However, the cultural difference in vertical individualism between the two groups of people was not significant, suggesting that they value competition, success, and achievements to the same extent. Moreover, Moroccan–Dutch even showed an unexpected higher degree of horizontal individualism, meaning they emphasized individual equality more than native Dutch. These unexpected results on cultural differences might be explained by acculturation, which means the immigrants’ individualism and collectivism are oriented to the host culture [41]. It is also important to mention that the Individualism–Collectivism Scale might not be the optimal measurement to test the cultural differences in the current research. Firstly, internal consistency problems were found on the four dimensions, with Cronbach’s α ranging from 0.47 to 0.74, which is rather low. Secondly, a principal component analysis demonstrated five principal components in the Individualism–Collectivism Scale, which contradicted the theoretical four-dimensional structure. Moreover, research has shown that Moroccan–Dutch and Turkish–Dutch transmitted their collectivism values to the next generation, but not individualism values [42]. This could indicate that collectivism might be a stronger cultural feature than individualism for these two immigrant groups, and might be relatively hard to dissimilate in the host culture. Given these reasons, a unidimensional collectivism scale was used to examine cultural differences in the second study.

A second study targeting native Dutch and Turkish–Dutch was conducted to further examine the proposed moderation effect of cultural background between persuasion and healthy eating intention. A prior sample analysis was done by G*Power [43]. Based on an estimation of statistical power of (1 − β) = 0.80 and effect size of η_p_^2^ = 0.07 obtained in Study 1, a minimum of 102 participants was required for detecting the proposed interaction between persuasion and cultural background. Native Dutch and Turkish–Dutch were equally assigned to the online and offline version. We aimed to replicate the interaction obtained from Study 1.

## 4. Study 2: Materials and Methods

Design. A 2 (persuasion: Self-persuasion vs. direct persuasion) × 2 (cultural background: Dutch vs. Turkish) between-subjects design was used, with persuasion and culture as factors, and healthy eating intention as the dependent variable.

Sample. The experiment was both programmed online in Qualtrics and printed out on paper. Native Dutch and Turkish–Dutch were invited to participate online through the study link distributed on Facebook pages and via the researchers’ social networks, and to participate offline in field locations such as Utrecht Central Station and typical Turkish teahouses. In total, 175 participants took part in the experiment. Of them, 36 participants were excluded before data analysis because they had participated in the same research before (*n* = 4), indicated their origin as neither the Netherlands or Turkey, (*n* = 16), did not strongly identify themselves with either of both cultures (*n* = 4), spent either more than 20 min or less than 2 min in doing the survey (indicating that they did not finish it in one go, or that they participated carelessly, *n* = 12), or scored 2.5*SD*s above or below the mean on the collectivism scale [41] (*n* = 4). The remaining 139 participants (*M*_age_ = 28.62, *SD*_age_ = 13.12; *n*_female_ = 68, *n*_male_ = 71) consisted of 69 native Dutch and 70 Turkish–Dutch. Of them, 32 native Dutch and 38 Turkish–Dutch participated online, whereas 37 native Dutch and 32 Turkish–Dutch participated offline (Moroccan–Dutch approached online and offline did not differ in self-reported healthy eating intentions (*p* = 0.624)) (see Table 3 for participants’ demographics). All participants participated voluntarily.

Procedure and materials. The procedure and materials (Uncertainty avoidance was measured for exploratory reasons) used in this study were identical to those in Study 1, except for the following parts.

Instead of the four-dimensional Individualism and Collectivism Scale [40], a unidimensional scale was used to access participants’ degree of collectivism [44]. Participants had to respond to five items (Cronbach’s α = 0.77) on a 5-point scale (1 = “very unimportant”, 5 = “very important”). A higher total score indicated a higher degree on collectivism and a lower degree on individualism.

Moreover, to collect more information about the cultural differences between native Dutch and Turkish–Dutch, all participants were asked to what extent they feel that they are Dutch (1 = “not at all”, 7 = “very much”). Also, the Inclusion of Other in the Self (IOS) Scale was added to measure perceived interpersonal connectedness [45]. This single-item pictorial scale provides seven pairs of circles—one circle labeled “self”, the second labeled “other”—ranging from two totally separate circles (1) to two almost entirely overlapping circles (7). Participants were asked to write down the number of the pair which best describes their interpersonal connectedness with others.

Finally, a question was added asking whether participants had taken part in the same research before, in order to filter out those who accidentally participated in both Study 1 and Study 2. After participation, all participants were thanked and debriefed face-to-face or via email.

## 5. Study 2: Results

The statistical analyses were conducted in SPSS 25. To check the proposed cultural differences between native Dutch and Turkish–Dutch, three independent *t*-tests were done between these two groups of participants on collectivism, perceived interpersonal connectedness, and the extent to which participants felt like they are Dutch. Results showed that Turkish–Dutch were more collectivistic than native Dutch, *t*(137) = 3.87, *p* < 0.001, *d* = 0.66. In addition, they felt less Dutch than native Dutch, *t*(99.38) = −9.00, *p* < 0.001, *d* = 1.52. The difference in perceived interpersonal connectedness did not reach significance, *t*(122.53) = 1.61, *p* = 0.111, *d* = 0.28 (see Table 4 for descriptive information).

Firstly, to examine whether version influenced the results, a one-way ANOVA with survey version (offline vs. online) as the factor and healthy eating intention as the dependent variable was performed. Unexpectedly, results showed a significant version effect on healthy eating intention, *F*(1,137) = 7.10, *p* = 0.009, η_p_^2^ = 0.05. Participants in the online version reported significant higher healthy eating intention (*M* = 14.67, *SD* = 4.77) than participants in the offline version (*M* = 12.41, *SD* = 5.25). Therefore, the version was included as an additional factor in the main analysis.

An ANOVA was conducted on healthy eating intention, with persuasion, cultural background, and version as factors. The main effect of version was significant, *F*(1,131) = 6.79, *p* = 0.010, η_p_^2^ = 0.05. Participants in the online version reported significant higher healthy eating intentions (*M* = 14.67, *SD* = 4.77) than participants in the offline version (*M* = 12.41, *SD* = 5.25). Results also showed a significant three-way interaction between persuasion, cultural background, and version, *F*(1,131) = 7.43, *p* = 0.007, η_p_^2^ = 0.05. All the other main effects and interactions did not reach significance (minimal *p* = 0.160, maximum η_p_^2^ = 0.02).

**Figure 2:**
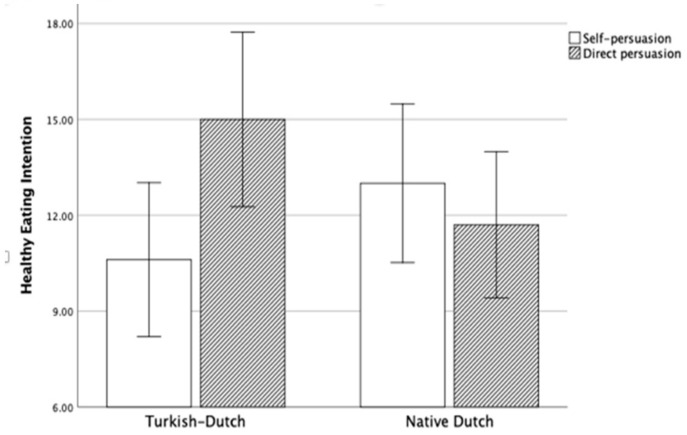
The interaction between persuasion and cultural background in the offline version in Study 2.

To break down the three-way interaction, the interaction between cultural background and healthy eating intention was analyzed separately for both survey versions. We found the interaction between cultural background and healthy eating intention significant in the offline version, *F*(1,65) = 5.23, *p* = 0.025, η_p_^2^ = 0.08 (see Figure 2), but not significant in the online version, *F*(1,66) = 2.38, *p* = 0.128, η_p_^2^ = 0.04. Subsequently, simple effect analysis of the offline version demonstrated that the sensitivity for self-persuasion and direct persuasion differed for Turkish–Dutch, but not for native Dutch. As expected, Turkish–Dutch participants in the direct persuasion condition reported significantly higher healthy eating intentions (*M* = 15.00, *SD* = 4.30) than in the self-persuasion condition (*M* = 10.61, *SD* = 5.10), *F*(1,65) = 5.79, *p* = 0.019, η_p_^2^ = 0.08. In the native Dutch participants, no significant difference between self-persuasion (*M* = 13.00, *SD* = 5.16) and direct persuasion (*M* = 11.70, *SD* = 5.60) was found, *F*(1, 65) = 0.59, *p* = 0.444, η_p_^2^ = 0.01.

A set of additional analyses were conducted to examine whether participants in the online and offline version differ from each other, as explorations for explaining the version effect outlined above. First, independent *t*-tests on participants’ demographics and cultural-related characters between the online and offline version of the experiment showed that participants in the offline version were significantly older (*t*(91.72) = −5.00, *p* < 0.001, *d* = 0.85; more collectivistic (*t*(137) = −2.96, *p* = 0.004, *d* = 0.50) and more interpersonally connected with others (*t*(137) = −2.19, *p* = 0.030, *d* = 0.37) than participants in the online version. Second, independent *t*-tests between native Dutch and Turkish–Dutch for the online and offline version separately showed that Turkish–Dutch scored significantly higher on collectivism (*t*(67) = 4.91, *p* <. 001, *d* = 1.18) and marginally significant higher on IOS (*t*(53.92) = 1.90, *p* = 0.063, *d* = 0.46) than native Dutch. However, in the online version, Turkish–Dutch and native Dutch did not show significant differences on both collectivism (*t*(68) = 1.63, *p* = 0.107) and IOS (*t*(66.22) = 0.64, *p* = 0.527).

In contrast to Study 1, a theoretically unexpected version effect was found on healthy eating intention, indicating that participants who participated online versus offline responded differently on the dependent variable. Including version as an additional factor, the proposed interaction was found significant only in the offline version, with Turkish–Dutch reporting higher intentions to eat healthily when exposed to direct persuasion compared to self-persuasion. However, the expected moderation effect was not present in the online version. This unexpected version effect could partly be due to participants of the two versions being significantly different from each other in terms of age, collectivism, and perceived interpersonal connectedness. Additionally, the native Dutch and Moroccan–Dutch online groups did not differ on the levels of collectivism and perceived interpersonal connectedness, which could have led to non-significant differences in the online version.

Regardless of versions, the two ethnic groups showed a significant cultural difference with Turkish–Dutch being more collectivistic than native Dutch, which is in line with previous literature suggested by Hofstede [35]. In addition, Turkish–Dutch felt that they were less Dutch compared to native Dutch, although, generally speaking, they felt more Dutch than not (*M* = 4.33 on a 7-point scale). This seems reasonable since most of them either grew up in the Netherlands or have resided there for many years.

## 6. Discussion

The current research investigated the moderation effect of cultural background in the relationship between persuasion and healthy eating intention among culturally diverse groups of native Dutch, Moroccan–Dutch, and Turkish–Dutch.

We found support for the moderation effect of cultural background in Study 1 as well as in the offline version in Study 2. Particularly, self-persuasion was found to be more effective than direct persuasion for native Dutch in Study 1, which is in line with previous findings on self-persuasion effect in other health domains [13,25]. Further, the finding that Turkish–Dutch benefited more from direct persuasion than self-persuasion in the offline version of Study 2 is consistent with our proposal that people from collectivistic cultures are more compliant to direct persuasion compared to self-persuasion [33,34]. Interestingly, in the offline version of Study 2, no significant effect between the two types of persuasion was found in the native Dutch group. This unexpected result might be related to the fluency of the self-generation process or participants’ different level of agency. Research has shown that self-persuasion can achieve its optimal effect when people are fluent with generating self-arguments [46], and it is less effective than direct persuasion even in Westerners if they have a low level of agency [37]. Both of these factors, or other unknown moderators, could be responsible for the absence of a self-persuasion effect in native Dutch. Furthermore, insufficient power may be another reason, considering the current results are based on parts of data which were obtained offline rather than the complete data.

As mentioned above, the moderation effect of cultural background was not found among participants in the online version in Study 2. Some may argue that the survey version might influence how people respond to persuasive attempts. However, it is made unlikely according to previous research findings in online and offline persuasion [47]. For example, Matheson and Zanna found that whether participants read the persuasive communication online or on paper did not influence their reported attitude [48]. Similar empirical evidence was also found by Hill and Monk, who showed that the persuasiveness of computer-mediated communication is equivalent to that of printed text [49]. In accordance, Wilson made the same claim that the differences between face-to-face communication and computer-mediated communication do not necessarily affect the overall persuasiveness of messages, and that these two modalities of communication could exert comparable persuasive effectiveness [50]. Hence, we claim that the missing moderation in the online version was unlikely due to the version itself. Instead, supported by our results showing no cultural differences between native Dutch and Turkish–Dutch in the online version, the missing moderation effect could to a large extent result from these two groups lacking variation in cultural orientations. It appears that Turkish–Dutch in the online version were well acculturated in the process of prolonged, continuous intercultural contact with Dutch culture. Age at which migration occurred, and the immigrants’ educational level were found to be factors that could largely influence the success of acculturation: Younger and better-educated immigrants were observed to be more willing and able to adapt to the host culture [51,52]. Supporting this theory, Turkish–Dutch in the online version were significantly younger and better educated than their Turkish–Dutch counterparts in the offline version. Accordingly, they were significantly less collectivistic than those in the offline version. Together with the fact that those younger and better-educated Turkish–Dutch were not different from native Dutch in terms of collectivism (results obtained in the online version), it suggests that the Turkish–Dutch in the online version had already assimilated to mainstream Dutch culture values. Since younger and better-educated people are generally more involved in online activities, especially visiting social network sites [53], it is reasonable to see the most acculturated Turkish–Dutch participated online rather than offline.

Although the results across studies are mixed, it should be noted that the moderation effect of cultural background was present in samples where cultural differences were salient. Taken together, results from the current research supports the notion that cultural background moderates the relationship between persuasion and healthy eating intention, provided that cultural differences are present in the participants. Native Dutch and immigrants were found to not necessarily differ in cultural orientation, which demonstrates the need for taking acculturation into account when considering relevant issues in the context of immigration.

The current research has several strengths and limitations. By recruiting immigrants with a different cultural background, we reached the groups of people who are oftentimes neglected by previous research, and our preliminary findings shed light on the application of persuasion on both ethnic majorities and minorities in the Netherlands. Methodologically, the use of a survey experiment enabled both internal and external validity by assembling the experimental components into a survey format [54,55]. However, due to resource constraints, the current research used convenient samples, which involved self-selection and might not sufficiently represent the target groups given that people who voluntarily participated may be more interested in the research question or more willing to help. As results suggested, participants’ demographics were different across versions in Study 2, creating a version effect that we did not expect. Additionally, considering the relatively large sample size needed, we were not able to include a control condition in which participants were presented with no information. Furthermore, participants were asked their healthy eating intention right after the poster exposure. In future research, a more ecological valid design could be used to assess long-term influences [56], examining participants’ healthy eating intention across time. Future research could also consider including a real behavioral measure, for example making participants choose from healthy low-calorie food or unhealthy high-calorie food as the compensation for participation.

In the current research, participants were told a cover story that the last part of the experiment wass a general health survey, and were provided three filler questions asking about their drinking intention in the upcoming month, followed by three questions measuring their healthy eating intention. Although there is no evidence in the current study for a confounding influence of the filler questions, more attention should be paid to avoid that the fillers confound participants’ responses on healthy eating intention in such an experimental design. Future research could therefore consider using a different approach, such as filler questions irrelevant to the health topic, to avoid the possible problem. Regarding the survey language, the questionnaire used in the current research was translated into Dutch, with the assumption that all Moroccan–Dutch and Turkish–Dutch participants could well understand it. However, we did not explicitly ask participants to report their language ability. As fluency could influence the effectiveness of self-persuasion [46], in future research, questions asking participants to which degree they could read and understand the instructions, background information about their first and foreign language abilities, and fluency experience should be included to control for possible confounding variables. Another important issue to note is that individuals’ motivation to change is closely related to behavioral change. Research has shown that people who are ready and motivated to change intentionally remind themselves of the positive aspects of the desired attitudes or behaviors [57]. Moreover, motivated people tend to seek information consistent with their desired attitudes and evaluate this information as more credible [58]. Given that during self-persuasion, people think about arguments to perform a certain behavior, it could be assumed that motivation increases the effectiveness of this influence technique. Therefore, in future research it would be highly relevant to investigate which role motivation plays in the effectiveness of self-persuasion.

The current study has both theoretical and practical implications. Theoretically, it is one of the first studies introducing culture as a potential moderator in (self-)persuasion literature. It extends the limited understanding of the self-persuasion effect, especially in correcting the impression that self-persuasion is a one-size-fits-all persuasive technique, and adds to the emerging literature showing that several boundary conditions are of influence [37,46,59]. Practically, we hope our results could inspire relevant health agencies with strategies of designing persuasive messages in mass media campaigns, not only targeting people with Western cultural backgrounds, but also more collectivist immigrant groups that are oftentimes not reached through conventional health campaigns.

## 7. Conclusions

To conclude, culture influences the effectiveness of different persuasion techniques. Self-persuasion appears to be more effective in promoting healthy eating intentions of people with individualistic backgrounds, and direct persuasion to be more effective in people with collectivistic backgrounds. In an immigration context, however, the immigrants’ cultural orientation should be taken into consideration before determining which technique is optimal, because immigrants may already have assimilated to the host culture and hold the corresponding cultural values. Nevertheless, the current research calls our attention to the importance of taking culture into account when tailoring health campaign messages.

## Figures and Tables

**Table 1:** Means and standard deviations for participants’ age and education in Study 1.

	Dutch & SP ^2^ (*n* = 26)		Dutch & DP ^3^ (*n* = 23)		Moroccan & SP (*n* = 24)		Moroccan & DP (*n* = 18)	
	*M*	*SD*	*M*	*SD*	*M*	*SD*	*M*	*SD*
Age	22.08	1.29	21.35	1.99	19.50	3.20	18.50	2.04
Education ^1^	8.58	0.86	7.57	1.67	6.37	1.53	6.33	1.57

^1^ Education was reported on a 9-point scale from Lager onderwijs (elementary school) to WO (research university). ^2^ SP: Self-persuasion, ^3^ DP: Direct persuasion.

**Table 2:** Means and standard deviations on cultural-related variables in Study 1.

	Native Dutch (*n* = 49)		Moroccan-Dutch (*n* = 42)	
	*M*	*SD*	*M*	*SD*
Horizontal collectivism	26.90	3.87	29.02	4.39
Vertical collectivism	22.27	4.03	30.14	5.06
Horizontal individualism	25.84	4.51	29.10	4.28
Vertical individualism	19.86	4.99	18.21	5.34

**Table 3:** Means and standard deviations for participants’ age and education in Study 2.

	Dutch & SP ^2^ (*n* = 34)		Dutch & DP ^3^ (*n* = 35)		Turkish & SP (*n* = 32)		Turkish & DP (*n* = 38)	
	*M*	*SD*	*M*	*SD*	*M*	*SD*	*M*	*SD*
Age	27.65	12.31	30.60	16.08	29.38	13.25	27.03	10.70
Education ^1^	8.35	1.01	8.31	0.72	6.77	1.75^†^	6.68	2.04

^1^ Education was reported on a 9-point scale from Lager onderwijs (elementary school) to WO (research university). ^2^ SP: Self-persuasion, ^3^ DP: Direct persuasion. ^†^ There was one missing value on education among Turkish–Dutch.

**Table 4:** Means and standard deviations on cultural-related variables in Study 2.

	Native Dutch (*n* = 69)		Turkish-Dutch (*n* = 70)	
	*M*	*SD*	*M*	*SD*
Collectivism	18.23	3.67	20.91	4.46
Perceived interpersonal connectedness	3.94	1.22	4.36	1.78
Feeling Dutch	6.33	0.80	4.34	1.67
